# Trends and cost structure of drug-based secondary prevention of ischemic strokes

**DOI:** 10.1186/s42466-024-00356-x

**Published:** 2025-01-02

**Authors:** Konstantin Kohlhase, Ferdinand O. Bohmann, Christian Grefkes, Adam Strzelczyk, Laurent M. Willems

**Affiliations:** 1 Goethe University Frankfurt, University Hospital, Department of Neurology, Schleusenweg 2-16, 60528 Frankfurt am Main, Germany; 2 Goethe University Frankfurt, University Hospital, Epilepsy Center Frankfurt Rhine-Main, Frankfurt am Main, Germany

**Keywords:** DOAC, DAPT, Platelet aggregation inhibition, DDD, Health-economic

## Abstract

**Background:**

Advances in secondary stroke prevention, including direct oral anticoagulants (DOACs), dual antiplatelet therapies (DAPT), and cardiovascular risk management, have changed costs over the past decade. This study aimed to evaluate annual treatment costs and trends in drug-based secondary prophylaxis after ischemic strokes.

**Methods:**

Annual treatment costs were evaluated using the net costs per defined daily dosage (DDD) of discharge medications for ischemic stroke patients treated in 2020 at the University Hospital Frankfurt, Germany. Evaluated drugs included acetylsalicylic acid, adenosine diphosphate inhibitors, DOACs, vitamin K antagonists, lipid-lowering drugs (LLD), antihypertensives (AHT), and oral antidiabetics (OD). Kruskal–Wallis test examined intergroup differences in substance groups and stroke etiologies. DDD development between 2004 and 2021 was further evaluated for significant trend changes using an interrupted time series analysis.

**Results:**

The study included 422 patients (70.5 ± 12.9 years, 43.1% female). Etiologies divided into large-artery atherosclerosis (29.9%), cardioembolic (25.6%), cryptogenic (26.8%), and small-vessel disease (17.8%). The total estimated annual drug expenditure was € 241,808; of which 51.6% was due to DOACs (median € 1157 [Q1–Q3:1157–1157], *p* < 0.006), 20.0% to AHTs (€127.8 [76.7–189.8]), 15.7% to ODs (€525.6 [76.7–641.5]), and 8.7% to LLDs (€43.8 [43.8–43.8]). Cardioembolic strokes had the highest annual costs per patient (€1328.6 [1169.0–1403.4]) with higher expenditure for DOACs (*p* < 0.001) and AHTs (*p* < 0.026). DAPT costs were highest for large-vessel strokes (*p* < 0.001) and accounted for 2.5% of total costs. There was a significant trend change in DDDs for clopidogrel in 2010 (*p* < 0.001), for prasugrel in 2017 (*p* < 0.001), for ASA in 2015 (*p* < 0.001) and for DOACs in 2012 (*p* = 0.017).

**Conclusions:**

DOACs for cardioembolic strokes were the primary cost driver in drug-based secondary stroke prevention, whereas permanent ASA and DAPT only accounted for a minor cost proportion. LLDs were associated with lower costs than AHTs and ODs. There were significant changes in DDDs for the respective substances, whereas the costs for DOACs as the most expensive pharmaceuticals remained widely stable across the last decade.

**Supplementary Information:**

The online version contains supplementary material available at 10.1186/s42466-024-00356-x.

## Introduction

The burden of ischemic strokes remained unchanged as the second leading cause of death and the third leading cause of permanent physical disability worldwide [1]. Although improvements in prevention and treatment have resulted in a decrease of death numbers for many years, mortality has recently risen again in parts of Europe [2]. In addition to the individual consequences for the patient and their environment, strokes also represent a relevant economic burden for healthcare systems in Germany and worldwide [3]. Although there are often high indirect costs due to transient or permanent disability, high direct medical costs arise from primary care and from the usually permanent indication for secondary prophylactic medication [4, 5]. Besides demographic changes, an increase in cardiovascular risk factors such as chronic nicotine abuse, arterial hypertension, diabetes mellitus, and hypercholesterolemia over the past decade have been revealed as possible causes [2, 6]. While primary prevention targets the occurrence of the first ischemic stroke and often has to be applied to a wide part of the population, secondary prevention offers a more focused and risk-adapted attempt [7]. Especially in secondary prevention after ischemic strokes, platelet aggregation inhibitors (PAIs) and oral anticoagulants (OACs) are cornerstones that are complemented by lipid-lowering drugs (LLDs), antihypertensives (AHTs) and oral antidiabetics (ODs) [8]. During the last two decades, drug based stroke prevention has changed. On the one hand, randomized-controlled trials have identified patient groups in whom an additional transient dual antiplatelet therapy (DAPT) is beneficial [9–12]. On the other hand, the introduction of direct oral anticoagulants (DOACs) has redefined the treatment of cardioembolic strokes with atrial fibrillation [13]. Consequently, treatment costs for secondary stroke prevention changed and became more specific depending on the underlying stroke etiology, cardiovascular risk profile and chosen treatment strategies. Reflecting the general cost trend of pharmaceutical spendings reaching an all-time high of EUR 274 billion in Germany in 2022, the identification and evaluation of particularly cost-intensive treatments is important for a healthcare system [14].

The aim of this study was to access the cost structure and expenditures for medical stroke prevention after an ischemic stroke as well as to analyze the net cost development and number of available preparations of frequently used drugs in secondary prophylaxis between 2004 and 2021.

## Material and methods

The cost calculation was based on the secondary prophylactic medication at discharge of patients, who were treated at the Department of Neurology of the University Hospital Frankfurt/Germany between January and December 2020 for an acute ischemic stroke (ICD-10 diagnosis I63.X) [15]. The University Hospital in Frankfurt is a trans-regional tertiary referral clinic in the Frankfurt/Rhine-Main metropolitan region in Germany, which comprised a total of 5.8 million inhabitants in 2020 [16]. Included stroke etiologies were small vessel disease, large vessel disease (≥ 50% symptomatic stenosis), cardioembolic, and cryptogenic strokes. The stroke etiologies were assessed from the discharge letter and were based on the TOAST classification [17]. Rare causes such as thrombophilia, dissections, vasculitic stroke, or paradoxical embolism in patent foramen ovale were excluded.

### Assessment of secondary prophylactic medication

Medical secondary prevention was divided into antithrombotics such as platelet aggregation inhibitors (PAIs) or oral anticoagulants (OACs) and pharmaceuticals for the treatment of cardiovascular risk factors, e.g., lipid-lowering drugs (LLDs), antihypertensives (AHTs) and oral antidiabetics (OD). PAIs included acetylsalicylic acid (ASA) and adenosine diphosphate (ADP) receptor inhibitors (i.e., clopidogrel, prasugrel, and ticagrelor). OACs were grouped into vitamin K antagonists (i.e.., phenprocoumon) and direct oral anticoagulants (DOACs) such as thrombin inhibitors (i.e., dabigatran) or factor Xa inhibitors (i.e., apixaban, rivaroxaban, edoxaban). LLDs included statins, acetidionones (i.e., ezetimibe), and fibrates. According to the National Care Guideline—Hypertension, AHTs comprised of first-line therapeutics such as ACE receptor inhibitors, AT-1 receptor antagonists, calcium channel blockers, and thiazides, as well as second-line therapeutics such as aldosterone receptor antagonists, loop diuretics, beta-blockers, alpha-receptor blockers, central alpha-2 receptor agonists, direct vasodilators, and renin inhibitors [18]. ODs included biguanides (i.e.., metformin), sulfonylureas, glinides, glitazones, DPP-4 inhibitors, and SGLT-2 inhibitors. Subcutaneous antidiabetic drugs (i.e., insulin or subcutaneous GLP-1 agonists) were not assessed.

### Cost calculation

Annual treatment costs of PAIs, OACs, LLDs, AHTs and ODs were calculated using the net costs of a defined daily dose (DDD) multiplied 365 days or the estimated duration of intake for transient DAPT. This method is established to calculate direct drug costs in neurological diseases following the bottom-up approach [19, 20]. The DDDs represent the average daily drug dosage for the treatment of a specific disease in an adult and are based on the calculations and data of the World Health Organization (WHO) and the GKV (German public health insurance) drug index of the Scientific Institute of the AOK (WIdO). The respective DDDs for 2020 were extracted from the published drug prescription reports from 2021 [21].

**Fig. 1:**
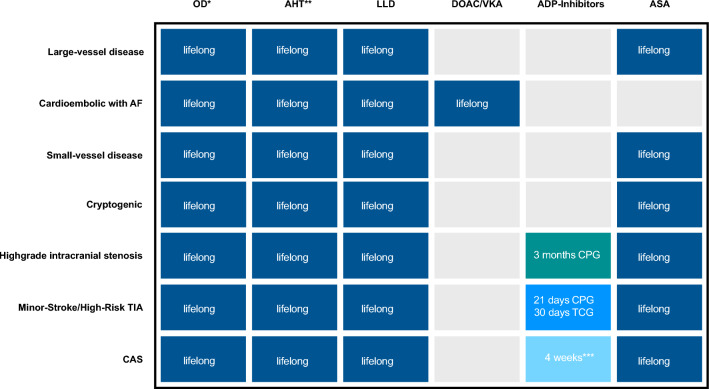
Secondary drug prophylaxis after ischemic stroke depending on the etiology (Large-vessel disease, small-vessel-disease, cardioembolic, and cryptogenic), as well as in selected cohorts with a high-risk stroke cause requiring transient dual platelet inhibition according to the German guidelines for secondary stroke prevention after ischemic strokes (e.g. high-grade intracranial stenosis, minor-stroke/high-grade transient ischemic attack, CAS). *AF *atrial fibrillation, *AHT* Antihypertensives, *ASA* Acetylsalicylic acid, *CAS* Carotid artery stenting, *CPG* Clopidogrel, *DOAC* Direct oral anticoagulants, *LLD* Lipid lowering drugs, *OD* oral antidiabetics, *TCG* Ticagrelor, *VKA* Vitamin K antagonists. *With proven diabetes mellitus, **With proven arterial hypertension, *** No standardized recommendation, usually individual assessment with at least 4 weeks

Antithrombotic treatment concepts were based on the recommendations of the German Society of Neurology (DGN) for secondary prophylaxis after acute ischemic stroke (2022) and the guideline for secondary prevention of symptomatic carotid stenosis of the German Society for Vascular Surgery and Vascular Medicine—Society for Operative, Endovascular and Preventive Vascular Medicine e.V. (DGG, 2020) [22, 23]. Transient treatment regimens with dual antiplatelet therapy (DAPT) included I. symptomatic high-grade intracranial stenoses (based on SAMMPRIS trial, 2011) [12], II. minor strokes or high-risk transient ischemic attacks (TIA) (based on CHANCE [2013], POINT [2018], and THALES trial [2020]) [10, 11, 24], III. stenting of the proximal internal carotid artery (CAS—carotid artery stenting) [23]. The respective treatment durations are displayed in Fig. 1.

### Cost development of PAIs and OACs between 2004 and 2021

The development of net DDD costs of ASA, ADP receptor inhibitors, VKA and DOACs between 2004 and 2021 were assessed and evaluated for significant trend changes using an interrupted time series analysis. Furthermore, the number of available preparations were assessed and contextualized to the development of DDDs between 2004 and 2021. The data were extracted from the published drug prescription reports (“Arzneiverordnungs-Report”) from 2005 to 2022 [25].

### Statistical analysis

Statistical analyses were performed using IBM SPSS Statistics software version 27.0.1.0 (IBM Corp., Armonk, NY, USA). The estimated yearly drug costs for the sub-groups “permanent PAI”, “permanent OAC”, “LLD”, “AHT” and “OD” were calculated using the net DDD in € multiplied with 365 days. For transient DAPT use, the treatment costs were calculated using the DDD multiplied with the duration of use based on the respective treatment guideline of the German Society of Neurology (DGN) for secondary prophylaxis after acute ischemic stroke (2022) and the guideline for secondary prevention of symptomatic carotid stenosis of the German Society for Vascular Surgery and Vascular Medicine—Society for Operative, Endovascular and Preventive Vascular Medicine e.V. (DGG) (2020) [22, 23]. The drug groups were tested for normal distribution using the Kolmogorov–Smirnov test. As normal distribution was rejected, median [First quartile–third quartile; Q_1_–Q_3_] was used as a primary measure of central tendency for the yearly drug costs of PAIs, OACs, LLDs, AHTs and ODs. Furthermore, mean ± standard deviation was reported as this represents an established value in cost analysis. A Kruskal–Wallis test was used to test for intergroup differences between substance groups and stroke etiologies. The cost development from 2004 to 2021 was analyzed for significant trend changes using an interrupted time series analysis (ITS) based on an independent correlation structure (Robust Interrupted Time Series Toolbox, Version 4.1.0, Biostatistics Research Group at the King Abdullah University of Science and Technology) [26]. A *p* value of < 0.05 was considered significant, all *p* values were Bonferroni-corrected.

## Results

### Prescription pattern and cost structure

In total 511 patients with ischemic strokes were treated in 2020. Of these, 59 patients were excluded from further analysis because of early death during hospitalization and another 30 patients due to ineligible stroke causes (Supplement Table 1). A total number of 422 patients (86.6%) were included in the final analysis. The mean age was 70.5 ± 12.9 years (range 30–99 years), with a share of 43.1% female patients. Stroke etiologies were divided into 29.9% (n = 126) large-vessel disease (LVD), 26.8% (n = 113) cryptogenic, 25.6% (n = 108) cardioembolic and 17.8% small-vessel disease (SVD) (n = 75).

#### Antithrombotics

Permanent PAI with ASA 100 mg was given in 71.8% (n = 303) and with clopidogrel 75 mg in 1.2% (n = 5). The indication for permanent monotherapy with clopidogrel 75 mg was ASA intolerance in all cases. Permanent treatment with prasugrel or ticagrelor was not recommended. OAC was recommended in 26.8% (n = 113), of which 69.0% (n = 78) were advised to use apixaban, 17.7% (n = 20) rivaroxaban, 3.5% (n = 4) edoxaban, 4.4% (n = 5) dabigatran, and 5.3% (n = 6) VKA (Table 1). The indication for OAC in all cases was atrial fibrillation. Transient PAI for minor stroke and high-risk TIA with clopidogrel 75 mg for 21 days analogous to the *CHANCE/POINT* trial was initiated in 12.3% (n = 52), treatment with ticagrelor for 30 days according to the *THALES* trial was not performed. For intracranial high-grade symptomatic stenoses, dual antiplatelet therapy analogous to the *SAMMPRIS* trial was recommended in 5.7% (n = 24). CAS with DAPT was prescribed in 5.4% (n = 23), of which 73.9% (n = 17) received clopidogrel 75 mg and 26.1% ticagrelor 2 × 90 mg (n = 6). DAPT after CAS was recommended for 3 months in 78.3% (n = 18), for 30 days in 4.3% (n = 1), and for 6 months in 8.7% (n = 2). Intracranial stenting was performed in 0.5% of patients (n = 2) with ticagrelor 2 × 90 mg for 3 months and lifelong ASA. Combined PAI and DOAC were initiated in 3.6% (n = 15) of whom 2 patients received triple therapy with ASA (lifelong), clopidogrel for 3 or 6 months and DOAC.

**Table 1:** The prescription frequencies and net DDD costs of drug-based stroke prophylaxis are presented for the investigated stroke collective

Medication			Patients [n = 422]	Percentage	DDD-Costs [€], 2020
Platelet aggregation inhibitors	COX-inhibitors	Acetylsalicylic acid	303	71.8	0.03
	ADP receptor/P2Y12 inhibitors	Clopidogrel	108	25.6	0.31
		Ticagrelor	9	2.1	2.50
		Prasugrel	1	0.2	1.89
Oral anticoagulants	Factor-Xa-Inhib	Apixaban	78	18.5	3.17
		Rivaroxaban	20	4.8	3.21
		Edoxaban	4	1.0	2.85
	Factor-II-Inhib	Dabigatran	5	1.2	3.62
	Vitamin-K-Ant	Phenprocoumon	6	1.4	0.2
Lipid-lowering drugs	Statin	Atorvastatin	354	83.9	0.12
		Simvastatin	34	8.1	0.18
		Rosuvastatin	9	2.1	0.13
		Pravastatin	2	0.5	0.19
		Fluvastatin	1	0.2	0.28
	Fibric acids	Fenofibrate	1	0.2	0.35
	2-Azetidinone	Ezetimibe	15	3.6	0.45
Antihypertensives	ACE-inhibitors	Ramipril	174	41.2	0.06
		Enalapril	7	1.7	0.09
		Lisinopril	3	0.7	0.1
	AT(1) receptor antagonist	Valsartan	23	5.5	0.12
		Candesartan	65	15.4	0.12
		Losartan	5	1.2	0.18
		Telmisartan	7	1.7	0.16
		Olmesartan	3	0.7	0.21
	Calcium channel blockers	Amlodipine	148	35.1	0.09
		Lercanidipine	29	6.9	0.1
	Thiazide diuretic	Hydrochlorothiazide	105	24.9	0.18
		Chlorthalidone	4	0.9	0.17
	Loop diuretic	Torasemide	72	17.1	0.19
	Beta-blocker	Bisoprolol	110	26.1	0.27
		Metoprolol	63	14.9	0.29
		Nebivolol	6	1.4	0.14
		Carvedilol	5	1.2	0.37
	Aldosterone Ant	Spironolactone	17	4.0	0.31
	Imidazole	Moxonidine	6	1.4	0.22
	Alpha blocker	Doxazosin	6	1.4	0.28
Oral antidiabetics	SGLT-2 inhibitors	Empagliflozin	19	4.5	2.08
		Dapagliflozin	3	0.7	1.27
	DPP-4 inhibitors	Sitagliptin	32	7.6	1.44
	Sulfonylurea	Glimepiride	2	0.5	0.14
	Biguanides	Metformin	71	16.8	0.21

Substances that were not used were removed from the table (Prasugrel, direct vasodilatators, renin-antagonists, glinids, glitazones)

#### Lipid-lowering drugs

LLDs were recommended in 95.7% (n = 404), subdivided into statins in 94.8% (n = 400), ezetimibe in 3.6% (n = 15), and fibrates in 0.2% (n = 1) of cases. Combined treatment with a statin and ezetimibe was given in 2.8% (n = 12). Atorvastatin was the most prescribed statin at 83.9% (n = 354), followed by simvastatin at 8.1% (n = 34) and rosuvastatin at 2.1% (n = 9) (Table 1).

#### Antihypertensives

AHT at discharge was given in 84.4% (n = 356), of which 43.6% (n = 184) received an ACE inhibitor, 24.4% (n = 103) an AT-1 antagonist, 17.1% (n = 72) loop diuretics, 43.6% (n = 184) beta-blockers, 25.6% (n = 108) thiazides, 41.9% (n = 177) calcium channel blockers, 4.0% (n = 17) aldosterone antagonists, and 2.8% (n = 12) sympatholytics such as moxonidine and doxazosin. Among ACE inhibitors, ramipril was prescribed most at 94.6% (n = 174), followed by enalapril at 3.8% (n = 7) and lisinopril at 1.6% (n = 3). For AT-1 antagonists, candesartan was recommended most often at 63.1% (n = 65), followed by valsartan at 22.3% (n = 23) and telmisartan at 6.8% (n = 7). Bisoprolol was the most prescribed beta-blocker at 59.8% (n = 110), followed by metoprolol at 34.2% (n = 63). Among calcium channel blockers, amlodipine was prescribed in 83.6% (n = 148) and lercanidipine in 16.4% (n = 29) (Table 1).

#### Antidiabetics

Oral antidiabetics were present in 20.4% (n = 86), of which an SGLT-2 inhibitor (empagliflozin or dapagliflozin) was taken in 25.6% (n = 22), sitagliptin in 37.2% (n = 32), glimepiride in 2.3% (n = 2), and metformin in 82.6% (n = 71) of cases (Table 1).

### Cost analysis

In the studied stroke population, the total estimated annual drug expenditure was € 241,808 corresponding to median treatment costs of € 240.9 [Q1–Q3: 124.1–1250.1; mean: € 560.4 ± 590.2 per patient. Of the annual total drug expenditures, permanent OACs accounted for € 124,888 in total or in median € 1157 [Q1–Q3: 1157–1157, mean: € 1131 ± 194.8] per treated patient (n = 113), whereas permanent PAI accounted for € 3883 in total or in median € 11.0 [Q1–Q3: 11.0–11.0; mean: € 12.6 ± 12.9] per treated patient (n = 308). The total cost of transient DAPTs were € 5317 for the respective treatment duration or in median € 28.3 [Q1–Q3: 6.5–28.3; mean: € 46.6 ± 104.2] per treated patient (n = 114). The total annual cost of LLDs were € 20,998 or in median € 43.8 [Q1–Q3: 43.8–43.8; mean: € 52.0 ± 31.5] per treated patient (n = 404) and for AHTs in total € 48,541 or € 127.8 [Q1–Q3: 76.7–189.8; mean: € 136.4 ± 77.3] in median per treated patient (n = 356). Oral antidiabetics accounted for € 38,179 or in median € 525.6 [Q1–Q3: 76.7–602.3; mean: € 443.9 ± 407.1] per treated patient (n = 86) per year. The results of Kruskal–Wallis-Test for intergroup differences and further details are reported in Table 2.

**Table 2:** The calculated costs of secondary preventive medication in a cohort of ischemic strokes [n = 422], categorized into permanent PAI, OAC, lipid-lowering drugs, antihypertensives, oral antidiabetics and transient DAPT are shown

Annual costs of secondary preventive medication in a cohort of ischemic strokes [n = 422]				
	N	DDD per patient in €, median [Q1–Q3]	Yearly costs per patient in €, median [Q1–Q3] and mean ± SD	∑ Yearly costs for the analyzed cohort
Permanent PAI	308	0.03 [0.03–0.03]	11.0* [11.0–11.0] 12.6 ± 12.9	€ 3883.6 [1.6%]
OAC	113	3.17 [3.17–3.17]	1157.1* [1157.1–1157.1] 1131 ± 194.8	€ 124,888.4 [51.6%]
Lipid-lowering drugs	404	0.12 [0.12–0.12]	43.8* [43.8–43.8] 52.0 ± 31.5	€ 20,998.5 [8.7%]
Antihypertensives	356	0.35 [0.21–0.52]	127.8* [76.7–189.8] 136.4 ± 77.3	€ 48,541.4% [20.0]
Oral antidiabetics	86	1.44 [0.21–1.76]	525.6* [76.7–641.5] 443.9 ± 407.1	€ 38,179.0 [15.7%]
Transient DAPT	114	–	28.3* [6.5–28.3] 46.6 ± 104.2	€ 5317.5 [2.5]
Total drug expenditure per year (incl. DAPT)	422	–	240.9 [124.1–1250.1] 560.4 ± 590.2	€ 241,808.4

The median [Q1–Q3] DDD net costs and yearly costs per patient in € as well as the cumulative annual treatment costs were calculated for the respective cohort. Intergroup differences were calculated using a Kruskal–Wallis test, level of significance was set at *p* < 0.05, significant values are marked in bold

*DAPT* Dual antiplatelet therapy, *DDD* Defined daily dose, *OAC* Oral anticoagulants, *PAI* Platelet aggregation inhibitors, *SD* Standard deviation

*Significant differences against every other drug type (*p* < 0.001), except “OAC versus oral antidiabetics (*p* = 0.005), no significant differences for “Permanent PAI versus Transient DAPT” (*p* = 0.16) and “Antihypertensives versus Oral Antidiabetics” (*p* = 0.053)

Subgroup analysis of stroke etiologies showed significantly higher costs of transient DAPT for LVD strokes with in median € 6.5 [Q1–Q3: 0.0–28.3, mean: € 36.4 ± 100.5] per patient (*p* < 0.001) compared to cardioembolic, SVD and cryptogenic strokes. Cardioembolic strokes significantly caused the highest treatment costs for OAC with in median € 1157 [Q3–Q3: 1157–1157; mean: € 895.2 ± 485.1] per patient per year (*p* < 0.001) as well as the highest total annual drug costs with a median of € 1328 [Q1–Q3: 1169–1403; mean € 1155.1 ± 575.7] per year (*p* < 0.011). Furthermore, AHT expenditure was significantly higher in cardioembolic strokes with in median € 136.9 [Q1–Q3: 88.5–202.6; mean: € 142.0 ± 84.3] per patient per year (*p* < 0.026); there were no significant differences between LLDs or ODs across etiologies (Table 3).

**Table 3:** Median [Q1–Q3] calculated annual costs of secondary preventive medication in a cohort of ischemic strokes, categorized into permanent PAI, permanent OAC, lipid-lowering-drugs, antihypertensives, oral antidiabetics and transient DAPT with regard to the underlying stroke etiology

	Cardioembolic (N = 108)	Large vessel disease (N = 126)	Small vessel disease (N = 75)	Cryptogenic (N = 113)
Transient DAPT	0.0 [0.0–0.0]	6.5** [0.0–28.3]	0.0 [0.0–6.5]	0.0 [0.0–0.0]
PAI	0.0** [0.0–0.0]	11.0 [11.0–11.0]	11.0 [11.0–11.0]	11.0 [11.0–11.0]
OAC	1157.1** [1040.3–1157.1]	0.0 [0.0–0.0]	0.0 [0.0–0.0]	0.0 [0.0–0.0]
LLD	44.0 [44.0–44.0]	44.0 [44.0–44.0]	44.0 [44.0–44.0]	44.0 [44.0–44.0]
AHT	136.9* [88.5–202.6]	87.6 [21.9–164.3]	98.6 [41.8–157.0]	98.6 [21.9–178.9]
Oral AD	0.0 [0.0–0.0]	0.0 [0.0–0.0]	0.0 [0.0–76.7]	0.0 [0.0–0.0]
Total	1328.6** [1169.0–1403.4]	178.9 [98.6–312.1]	186.2 [109.5–317.6]	189.8 [76.7–299.3]

Intergroup differences were calculated using a Kruskal–Wallis test; level of significance was set *p* < 0.05 (significant values are marked in bold: * < 0.026, ** < 0.001)

*AD* Antidiabetics, *AHT* Antihypertensives, *DAPT* Dual antiplatelet therapy, *LLD* Lipidlowering drugs, *OAC* Oral anticoagulants, *PAI* Platelet aggregation inhibitors

### Cost development of antithrombotics between 2004 and 2021

**Fig. 2:**
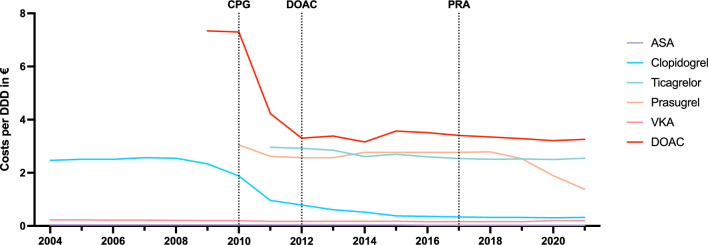
Cost development of DDD net costs over the years 2004 to 2021 for ASA, clopidogrel, ticagrelor, prasugrel, VKA and DOACs (averaged for Apixaban, Edoxaban, Rivaroxaban, and Dabigatran). Interrupted time series analysis was performed for significant trend changes and visualized by a dotted line for the respective drug type. *ASA* Acetylsalicylic acid, *DDD* Defined daily dose, *DOAC* Direct oral anticoagulant, *VKA* Vitamin K antagonist

**Fig. 3:**
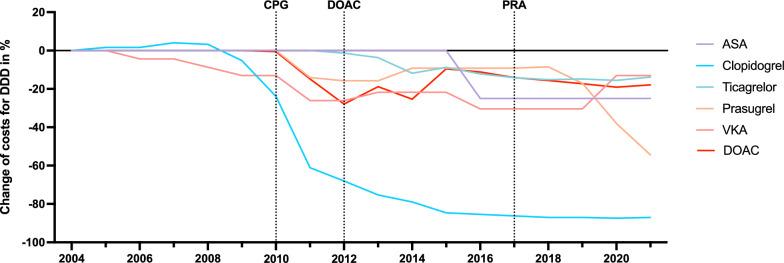
Percentual change of DDD net costs from 2004 to 2021 for ASA, clopidogrel, ticagrelor, prasugrel, VKA and DOACs (averaged for Apixaban, Edoxaban, Rivaroxaban, Dabigatran). *ASA* Acetylsalicylic acid, *DDD* Defined daily dose, *DOAC* Direct oral anticoagulant, *VKA* Vitamin K antagonist

**Fig. 4:**
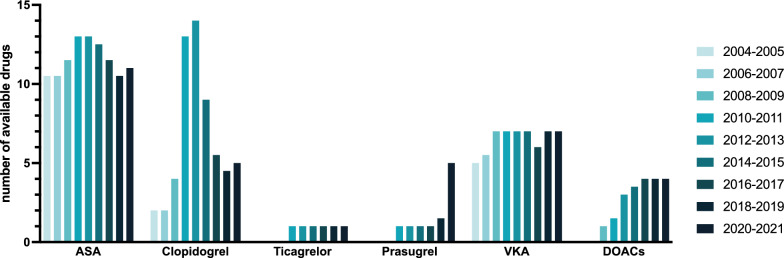
Number of available preparations for ASA, clopidogrel, ticagrelor, prasugrel, VKA and DOACs (in total for: Apixaban, Edoxaban, Rivaroxaban, Dabigatran) from 2004 to 2021. *ASA* Acetylsalicylic acid, *DOAC* Direct oral anticoagulant, *VKA* Vitamin K antagonist

Interrupted time-serial analysis revealed a significant change point for clopidogrel in 2010 (*p* < 0.001), for prasugrel in 2017 (*p* < 0.001), for ASA in 2015 (*p* < 0.001) and for DOACs (combined) in 2012 (*p* = 0.017). There were no significant trend changes for VKA or ticagrelor. The respective DDD development and change points were visualized in Fig. 2 with the percentual DDD change in Fig. 3 and number of available preparations in Fig. 4.

## Discussion

The present analysis of drug-based secondary prophylaxis after an ischemic stroke yielded significant differences in the annual costs for antithrombotics and the adjustment of cardiovascular risk factors, which was further depending on the underlying stroke etiology.

Among antithrombotics, DOACs and ADP inhibitors such as ticagrelor and prasugrel had the highest net DDD costs, whereby with 51.6% of the total annual drug costs in the examined stroke cohort, DOACs represented by far the largest cost factor in view of the greater prescription frequency and the usually lifelong use in cardioembolic strokes (Table 2). Although net DDD costs of VKAs are significantly lower than DOACs, they are rarely used in practice and limited to selected patients [27]. In the early benefit assessment of apixaban, as the most prescribed DOAC in the evaluated stroke cohort, the Federal Joint-Committee (G-BA) identified a minor additional benefit for the prophylaxis of ischemic strokes in atrial fibrillation compared to VKA [28]. In addition, a recent meta-analysis for the prevention of strokes and systemic embolisms showed a reduction in major bleeding complications and all-cause mortality for DOACs with a comparable reduction in strokes and systemic embolisms [29]. The higher costs of DOACs must be weighed against the avoided direct, indirect and intangible expenses due to prevented complications such as (intracerebral) hemorrhages or recurrent strokes [30]. The estimated costs for high income countries in 2020 were $ 32,982 for intracerebral hemorrhages and $ 25,569 for ischemic strokes per patient per year [30]. Health economic evaluations have therefore demonstrated a positive cost-effectiveness for all DOACs compared to VKA in high-income countries [31, 32]. In this regard, DOACs legitimately represent the drug of first choice for secondary prevention after a stroke, which is also reflected in the number of prescriptions and the associated expenses. In 2021, apixaban has become the top-selling drug for statutory health insurances in Germany across all indications [33]. From 2004 to 2021, the net DDD costs of DOACs constantly exceeded those of ADP inhibitors, ASA or VKA (Fig. 2). Following the introduction of rivaroxaban in 2008 as the first DOAC, the interrupted time series analysis showed a significant reduction in costs in 2012 with mostly stagnating net DDD costs since then. A possible cause of the cost reduction from 2012 may be a consequence of the early benefit assessment of the AMNOG (2011), which resulted in a convergence of costs after the introduction of comparable factor Xa inhibitors [34]. Due to the ongoing patent protection of DOACs, a further reduction of DOAC costs has not yet been achieved. The cost development after the expiry of the supplementary protection certificate (SPC) and the marketing of the first generics will be decisive, as this will presumably lead to a significant reduction in costs [35]. A comparable trend was seen for clopidogrel with a significant trend change in 2010 and most recently for prasugrel from 2017, which correlated with the increasing number of available preparations (Figs. 3, 4). In contrast, the costs of ticagrelor remained mostly constant.

ADP inhibitors were used in secondary stroke prophylaxis, with a few exceptions in cases of ASA intolerance, exclusively as transient DAPT after minor stroke, high-risk TIA, high-grade intracranial stenosis or after CAS. Among them, clopidogrel was the most frequently prescribed drug, followed by ticagrelor, whereas prasugrel was not used in daily practice (Table 1). The emerging use of transient DAPT regimen in high-risk populations did not account for a relevant cost factor (2.5%) in the examined stroke cohort and was lower than the annual costs of LLDs, AHTs or oral antidiabetics. The treatment costs of transient DAPT were significantly highest for large vessel infarcts, for which most of the established treatment concepts were aimed at (i.e., SAMMPRIS, CAS) (Fig. 1) [12, 23]. In addition, it is assumed that also minor strokes and high-risk TIAs due to LVD particularly benefit from transient DAPT, so that a selection by the treating physicians is possible [22].

An interesting group are cryptogenic strokes, which also include embolic strokes of undetermined sources (ESUS, approx. 9–25% of all strokes) and in which a not confirmed atrial fibrillation is suspected as one of the main cause of stroke [36, 37]. In ESUS, intensified cardiac rhythm recording, e.g. using a loop recorder, was reported to detect atrial fibrillation in up to 41.4% within the first 3 years, which resulted in permanent OAC in 84% of these cases [38]. This subsequently influences further treatment costs and increases the proportion of patients who are reliant on long-term oral anticoagulation, particularly DOACs. Based on the aforementioned rate of atrial fibrillation in ESUS, approximately 47 patients of our collective would been diagnosed with atrial fibrillation within 3 years [38]. Assuming that these patients are put on a DOAC, this would mean additional costs of € 48,377.1 to € 61,586.5 per year. However, since the proportion of ESUS in cryptogenic strokes was not analyzed in our study an exact calculation and cost prediction for these cases was not possible.

For pharmaceuticals to treat cardiovascular risk factors, LLDs accounted for 8.7%, AHTs for 20.0% and oral antidiabetics for 15.7% of annual treatment costs, totaling 44.4% (Table 2). The net DDD costs of first choice AHTs (ACE inhibitors, AT-1 antagonists, thiazides, calcium channel blockers) were comparable or lower than the net DDD costs of the most frequently prescribed statins (Table 1), thus the higher total AHT treatment costs primarily were driven by combination therapies. In contrast, the net DDD costs for ODs such as DPP-4 inhibitors (€ 1.44) and SGLT-2 inhibitors (€ 1.27 and € 2.08) were higher than those of AHTs and LLDs; only the older preparations such as metformin (€ 0.21) and glimepiride (€ 0.14) showed comparable net DDD costs. Across different stroke etiologies, there were no significant differences in costs of LLDs or ODDs. However, there were significantly higher treatment costs for AHTs in cardioembolic strokes, which could be explained by the high co-incidence of arterial hypertension and atrial fibrillation as well as the often-combined frequency modulation treatment with a beta blocker [39].

### Limitations

This study has as few limitations, including the analysis of only one stroke cohort from a tertiary referral hospital, which carries a risk of drug selection bias, e.g., in the choice of drug within a substance group, or the common use of new treatment concepts such as transient DAPTs. However, due to the overall close proximity of DDDs within a substance class and the alignment with current guidelines, this variance is considered negligible. Furthermore, as a tertiary referral hospital, large vessel occlusions are transferred for mechanical recanalization so patients with cardioembolic strokes due to atrial fibrillation or LVD might be over-represented [40–42]. In addition, the evaluated period (2020) was influenced by the COVID pandemic, which had major impact on all health care sectors in Germany [43, 44]. During this period, stroke hospitalizations in Germany decreased by up to 10.9%, which also resulted in a lower number of treated patients with ischemic strokes [45]. In the evaluation, this particularly affects the expected total annual costs, which could be underestimated as a result, although the cost relations between the drug categories and etiologies were barely affected by this. In this regard, health data often shows a positive skewness, which can be enhanced by the inclusion of untreated patients and the use of averaged DDDs. As a result, the calculated pharmaceutical costs with their median and Q1–Q3 should only be interpreted as a comparative measure and not as an absolute value. Finally, it should be mentioned that our analysis cannot make any statement about drug compliance, which can reduce the actual treatment costs in the further course. In addition, the costs can change due to a modification of drug treatment in outpatient aftercare.

## Conclusion

The annual costs of secondary prophylaxis after an ischemic stroke are relevant in terms of health economics and depend on the etiology and the treatment of cardiovascular risk factors. While the drug costs for LLDs and ODs were comparable across all etiologies, there were significantly higher costs for AHTs in cardioembolic strokes. Furthermore, the use of DOACs in cardioembolic strokes accounted by far for the highest costs in drug-based secondary stroke prevention, whereas the use of permanent PAI with ASA or the increasingly implemented transient DAPTs in high-risk populations did not account for a relevant cost factor. While the costs of DOACs have recently remained mainly constant and exceeded those of other antithrombotics by far, there has been a reduction in the costs of ADP inhibitors such as clopidogrel, which may have been caused by healthcare reforms, expiring patent protection and the availability of different preparations. In view of the continuous pharmaceutical progress, the resulting costs always must be balanced against the health economic benefits. For this purpose, the presented data aimed to evaluate the cost distribution of medical stroke prevention in a representative stroke cohort and to summarize the recent cost-development of the most important antithrombotics.

## Supplementary Information

Additional file1

## Data Availability

The data analyzed in this study is subject to the following licenses/restrictions: data will be made available upon reasonable request due to German regulations on data protection. Requests to access these datasets should be directed to Konstantin Kohlhase, email: kohlhase@med.uni-frankfurt.de.

## Abbreviations

ASA: Acetylsalicylic acid
ADP: Adenosine diphosphate
AHT: Antihypertensive
DDD: Defined daily dose
DOACs: Direct oral anticoagulants
DAPT: Dual antiplatelet therapies
GKV: German public health insurance
LLD: Lipid-lowering drug
OD: Oral antidiabetics
OAC: Oral anticoagulant
PAI: Platelet aggregation inhibitor
VKA: Vitamin-K-antagonist
WHO: World Health Organization
