# Supplementary material for: Trends and cost structure of drug-based secondary prevention of ischemic strokes
Source: Neurol Res Pract. 2025 Jan 2;7:1. doi: 10.1186/s42466-024-00356-x (PMC11697035; doi:10.1186/s42466-024-00356-x)
Supplement: Supplementary file 1 — Additional file1 [file 42466_2024_356_MOESM1_ESM.docx]

**Supplementary Tables:**

| **Exclusion of ineligible stroke causes** | **Number of Patients** |
| --- | --- |
| Patent foramen ovale (PFO) | 8 |
| Dissection | 3 |
| Idiopathic intracranial stenosis arteriopathy | 1 |
| Periinterventional | 5 |
| Vasculitis | 3 |
| Coagulopathy | 1 |
| Valve prothesis without atrial fibrillation | 1 |
| Palliative Care | 2 |
| Sinus thrombosis | 1 |
| Incomplete Data | 5 |

Supplement Table 1: The table shows the distribution of excluded patients due to inegilible stroke causes.
